# Spectroscopic and viscometric determination of DNA-binding modes of some bioactive dibenzodioxins and phenazines

**DOI:** 10.1016/j.bbrep.2019.100629

**Published:** 2019-04-04

**Authors:** Apeksha Ashok Phadte, Subhadeep Banerjee, Nayan Anand Mate, Arnab Banerjee

**Affiliations:** a Department of Chemistry, BITS Pilani KK Birla Goa Campus, Zuarinagar, Goa, 403726, India; b Department of Biological Sciences, BITS Pilani KK Birla Goa Campus, Zuarinagar, Goa, 403726, India

**Keywords:** Intercalation, Minor groove binding, Dibenzodioxins, Phenazines, Push-pull

## Abstract

Push-pull dibenzodioxins and phenazines having ‘anthracene-like’ planar structures and good charge transfer character had been previously synthesised in our laboratory. The dibenzodioxins had earlier proven their anti-proliferative nature against HeLa tumor cell lines. Since phenazines are structural analogues of the former, these molecules were evaluated in course of the current study for their cytotoxic action against HeLa cell lines and they exhibited strong anti-tumor activity. This behavior could be related to their good DNA binding property. The DNA binding modes of molecules **1**–**4** (Fig. 1) were evaluated using various experimental techniques and they interacted with DNA in a non-covalently by both intercalative as well as groove binding mechanisms. Molecule **1** follows predominantly intercalative binding mode whereas molecules **2** and **3** have nearly equal and opposite preferences for both groove binding and intercalative modes. For molecule **4**, groove binding is preferred mode of binding to DNA. A rationale for such differential binding behaviour is provided based on the subtle structural differences in our synthesised dibenzodioxins and phenazines. Elucidation of the mode of a molecule-DNA-binding event is relevant for understanding the mechanism of action of these molecules and will help promote further research into designing better DNA targeting small molecules.

## Highlights

• DNA binding modes of push-pull planar dibenzodioxins and phenazines were elucidated.
• DNA binding mechanistic details were obtained by spectroscopic and viscometric techniques.
• The molecular shape and geometry has a bearing on its choice of binding mode.

## 1 Introduction

**Fig. 1:**
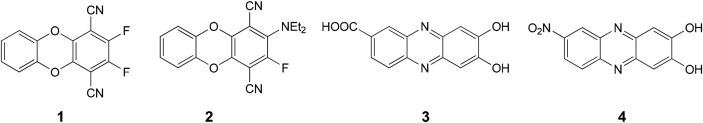
Examples of push-pull 1, 4-dicyanodibenzodioxins (**1** & **2**) and phenazine (**3** & **4**).

Planar ‘anthracene-like’ molecules exhibit strong anti-tumor activity arising from their good DNA binding property [[1], a), b)]. However, in drug development studies polycyclic aromatic hydrocarbons (PAHs) are unsuitable due to their general toxicity towards biological tissue [2]. Hence, structural analogues of PAHs could be used as platforms for designing small molecular drugs. We have focused our research on design and synthesis of push-pull dibenzodioxins and phenazines as attractive options due to their planar ‘anthracene-like’ structures and well established bioactivity [3,4]. We previously synthesised push-pull dibenzodioxin molecules (Fig. 1), and they exhibited promising *in vitro* cytotoxicity against HeLa tumor cell line and no cytotoxicity against normal HEK 293 and HaCaT cell lines [5,6]. The IC_50_ values of all derivatives against HeLa cell line were in low micromolar ranges and these values are similar as compared with earlier reported dibenzodioxins [7]. Since, phenazines have structural resemblance with dibenzodioxins, we are also interested in exploring their *in vitro* cytotoxicity against suitable tumor cell lines.

Also as a subset of bioactivity studies, researchers have focused on the DNA binding behaviour of small molecules since DNA is one of the main targets of anticancer drugs [8]. In order to investigate the molecule-DNA interactions, it is important to find their DNA-binding mode. DNA has different binding modes for non-covalent interactions with small molecules, where intercalation and minor groove binding are the most common ones [9]. Intercalation implies stacking insertion of a planar molecule between the layers of stacked bases in double-stranded DNA. While it does not directly damage DNA, but the DNA-intercalator complex inhibits the activity of topoisomerase enzymes involved in DNA replication processes [10]. Intercalation reduces DNA helical twist and lengthens the DNA [11]. Many small molecule intercalators exist among which Ethidium Bromide (EB) is a well-known example that binds tightly to DNA and is used to tag DNA in different biological experiments due to its strongly fluorescent nature [12]. In contrast to intercalation, groove binding does not alter DNA conformations, but the small molecule just sits along the minor groove of the DNA and is stabilized by H-bonding and Van der Waals interactions with the basic residues of the groove. The new ligand ends up occupying the place of water molecules along the groove [13]. Hence, such interaction is entropically favourable. 4′,6-diamidino-2-phenylindole (DAPI) is a minor grove binder that bind to A-T rich region in DNA and is also used as DNA tag due to its fluorescent nature [14]. A single compound may utilise more than one mode of DNA binding (e.g., intercalation and groove binding) [15]. Hence in addition to exploring the bioactivity of our prepared phenazines (Fig. 1), we are also interested to study the DNA binding mechanism for the same and also study details of DNA binding by our bioactive dibenzodioxins (Fig. 1). Different experimental techniques could be utilized for studying the drug-DNA binding interactions and distinguish the binding modes (intercalation from groove binding). UV–visible spectroscopy, fluorescence spectroscopy, and viscometry are among the principal techniques used. We here will also utilise all of these methods to study binding mode for our molecules with DNA.

## 2 Experimental

### 2.1 Materials and methods

All chemicals were reagent grade, purchased from commercial vendors. They were used as purchased. Calf thymus DNA (ct-DNA) was obtained from Sigma Aldrich, UV–Vis JASCO V-770 spectrophotometer was employed to check DNA purity (A260: A280 > 1.80) and concentration (€ = 6600 M^−1^ cm^−1^ at 260 nm) [16]. Interactions of the compounds with ct-DNA were studied using solutions of the compound in DMSO and ct-DNA in Tris–HCl buffer (pH 7.2) containing 5 mM Tris–HCl. The buffer solution was prepared with double-distilled water.

#### 2.1.1 (4,5-Dimethylthiazol-2-yl)-2,5-diphenyltetrazolium bromide (MTT) assay

This was carried out to evaluate the anti-proliferative activity against HeLa cells of prepared phenazine molecules (Fig. 1 molecule **3** and **4**). HeLa cells were plated on 96 well plates at density of 4*10^4^ per well and incubated with our compounds in concentrations of 2, 10 and 50 μM. After certain intervals, media was aspirated and fresh media with MTT (5 mg/ml) added. After 4 h, the MTT solution was removed and 100 μL of DMSO was added. Absorbance of the colored solution was measured at 570 nm with a reference at wavelength of 620 nm. The absorbance obtained from compound treated cells was always a fraction of absorbance obtained from untreated cells [17].

#### 2.1.2 Singlet oxygen quenching

Singlet oxygen quantum yields for our synthesised molecules (**1**–**4**) were measured relative to TPP (tetraphenyl porhyrin) using 1,3-diphenylisobenzofuran (DPBF) as a probe for singlet oxygen. Stock solutions of the respective compounds and the TPP were made in DMF with optical densities equal to 0.1 at their λ_max_ and Soret band respectively. Additionally a stock solution of the DPBF was prepared in DMF, such that 50 μL of this solution in DMF (3.0 mL) gave an optical density of 0.1 at 414 nm. To fluorescence cuvette was added the compound solution (3.0 mL) and the DPBF solution (50 μL) was mixed in. The solution in the cuvette was irradiated in 10 s intervals by projecting white light from a small torch of radius 1 cm held at fixed distance of 4 cm above the cuvette. The time dependent decrease in fluorescence of the DPBF (excitation 414 nm and emission 456 nm, 480 nm) was monitored. A separate blank was also run to monitor the bleaching of DPBF without any compound or TPP by using the DPBF solution (50 μL) in DMF (3.0 mL) [18]. The relative singlet oxygen quantum yield was calculated by comparing the slopes of different plots generated in Fig. 3.

#### 2.1.3 UV–Vis measurements

UV absorption spectra of ct-DNA were recorded on JASCO V-770 UV–visible spectrophotometer in the absence and presence of compounds at 298 K in the wavelength range of 250–700 nm. The quartz cuvette with 1 cm path length was used.

#### 2.1.4 Fluorescence based competitive dye displacement assay

The fluorescence spectra were measured on JASCO FP-8500 spectrofluorimeter. The excitation wavelengths were 526 nm and 358 nm for EB and DAPI respectively and emission recorded between 450 and 650 nm. The excitation and emission slit widths were maintained at 2.5 nm each. The fluorescence spectrum of ct-DNA was recorded at 298 K in presence of DAPI and EB in 5 × 10^−5^ M and 3.03 × 10^−4^ M concentrations respectively. Competitive dye displacement assay was carried out by adding compounds **1**–**4** in different concentrations. The DAPI–ct-DNA complex and EB–ct-DNA complex were titrated with increasing concentration of compounds from 0 to 50 μM range.

#### 2.1.5 Viscosity measurements

To determine the binding mode of compounds (**1**–**4**), viscosity measurements were performed by keeping the ct-DNA concentration constant (0.5 mM) and varying the concentration of compounds. Viscosity experiments were carried out using a Brookfield DV1 viscometer at 25 °C. The data were presented as (η/η_0_)^1/3^ versus [compound]/[DNA] ratio, where η and η_0_ are the viscosity of DNA in the presence and absence of compounds respectively.

## 3 Results and discussion

### 3.1 Evaluation of bioactivity of our phenazines

**Fig. 2:**
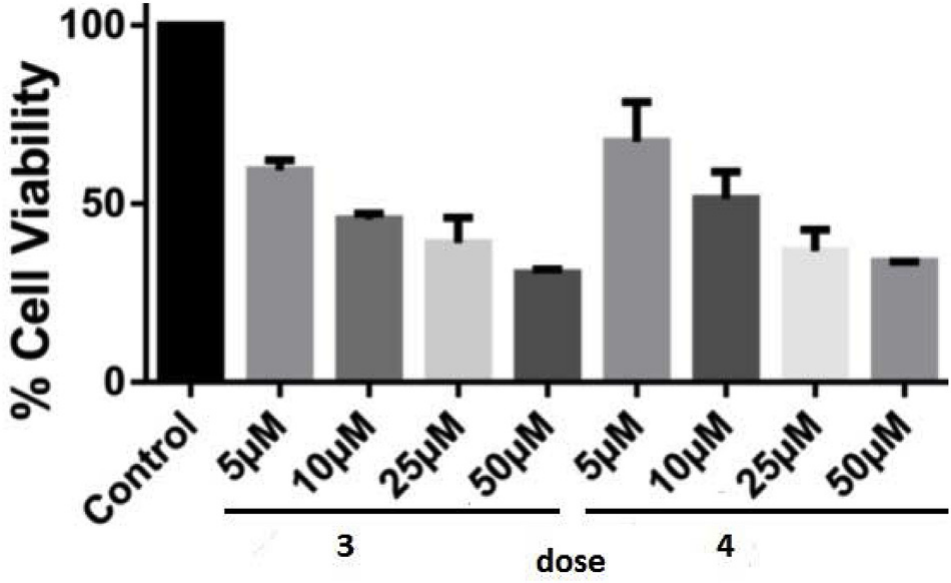
Effect of different doses of phenazines on the cell viability of cultured HeLa cells using MTT assay. HeLa cells were incubated with phenazines for 24 h. The vertical bars represent cell viability for each drug concentration ranging from 5 to 50 μM as means of three experiments (all doses are significant with p = 0.0001).

The cytotoxic behaviour of dibenzodioxin towards various tumor cell lines was well reported [7]. Since our synthesised phenazines have structural similarity with dibenzodioxins, we evaluated the anti-proliferative action of compounds **3** & **4** against human cervical cancer HeLa cell line. HeLa cells were cultured and incubated phenazine compounds **3** & **4** for 24 h and the cell viability was evaluated using MTT assay [17]. The results displayed that both the compounds **3** & **4** exhibit reduction of cell viability of the cultured HeLa cells (Fig. 2) measured against a control (cells treated only with DMSO). Both the molecules were active against HeLa cells among which molecule **3** was found to reduce cell viability up to 30% of control in 5–50 μM doses whereas molecule **4** reduced cell viability up to 33%. The findings thus further validated our idea that since phenazines are structurally similar to the dibenzodioxins, they could exhibit similar bioactivity profile against tumor cell lines.

### 3.2 Reactive oxygen species (ROS) production efficacy by dibenzodioxins and phenazines

**Fig. 3:**
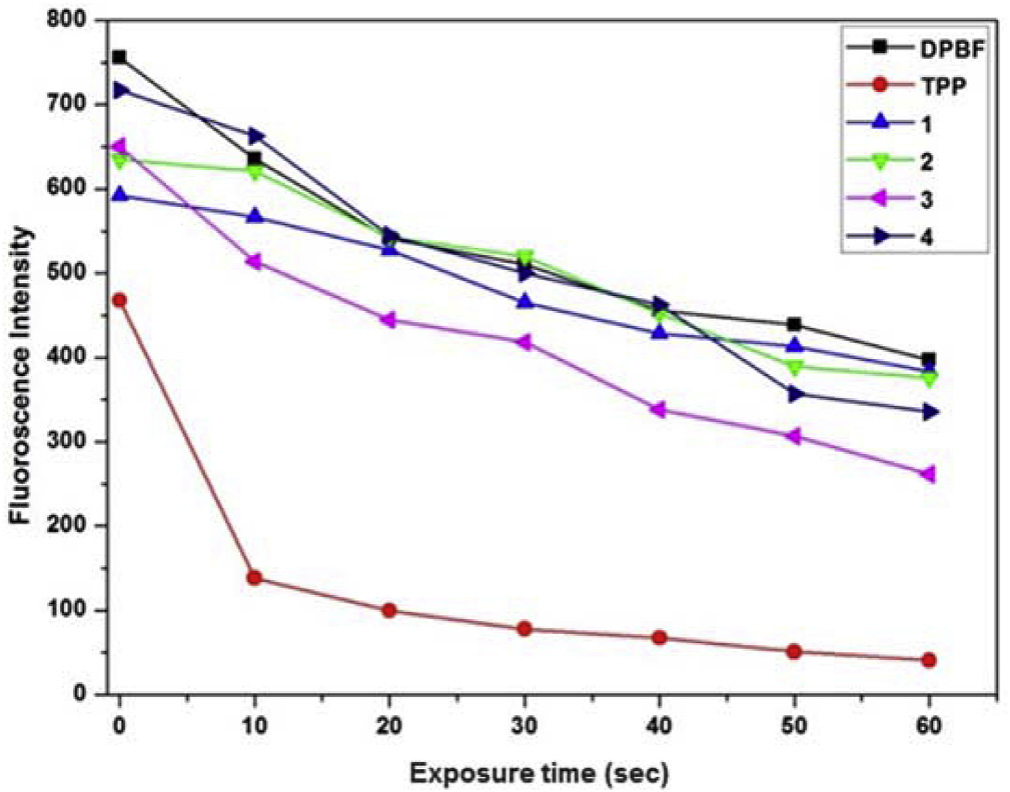
Time dependent fluorescence decay of DPBF in absence and presence of our molecules (**1**–**4**). Solution of DPBF and molecules in DMF were irradiated at room temperature with white light torch held at fixed distance of 4 cm above the cuvette. Average fluorescence emission at 458 nm and 480 nm was monitored as a function of time.

We have previously shown that HeLa cells incubated with our dibenzodioxins (compound **2** from Fig. 1 among others) suffered from oxidative stress condition [5]. When hypoxic tumor cells undergo drug induced life cycle changes, the levels of intracellular reactive oxygen species (ROS) increases. Beyond a certain threshold, ROS could activate programmed cell death pathways such as apoptosis [5]. Singlet oxygen (^1^O_2_), a form of ROS with very short half-life is also known to be very cytotoxic [18] and can be utilized in promoting tumor cell death by causing damage to cellular substructures, particularly the nuclear material. Based upon our previous observation of high level of ROS induction by dibenzodioxins (compound **2**) *in vitro*, [5] we were thus interested in evaluating whether and to what extent ROS could be produced by our dibenzodioxins and phenazines (Fig. 1) in solution. Whether there could be a connection between ROS generation and their bioactivity. Singlet oxygen quantum yields for our synthesised molecules (**1**–**4**) were measured relative to *mes*o-tetraphenylporphyrin (TPP) using 1,3-diphenylisobenzofuran (DPBF) as a probe for singlet oxygen [18]. Comparative data was obtained from a plot of decreasing DPBF fluorescence over time (Fig. 3) for a photosensitized mixture of DPBF and our compounds/TPP. Evaluation of the rates of DBPF fluorescence decrease suggested that our synthesised molecules **1**–**4** generated ^1^O_2_ in 11%, 12%, 18% and 19% respectively relative to TPP, demonstrating that they have at best only modest ability to produce ROS in solution. These values were calculated by comparing the slopes (Fig. 3) of the different compounds and TPP plots with the DPBF plot, first by normalizing with respect to DPBF and then again normalizing with respect to the slope of the TPP line. Therefore, our earlier observation of high *in vitro* ROS levels for incubated HeLa cells, [5] could be related to the cellular DNA-targeting by our cytotoxic dibenzodoxins, rendering the normal cellular processes inoperative and subsequently producing oxidative stress on the cell. Therefore, in order to understand the observed bioactivity of our molecules, it will be crucial to further study their DNA-binding mechanisms.

### 3.3 Determination of the DNA binding constants

**Fig. 4:**
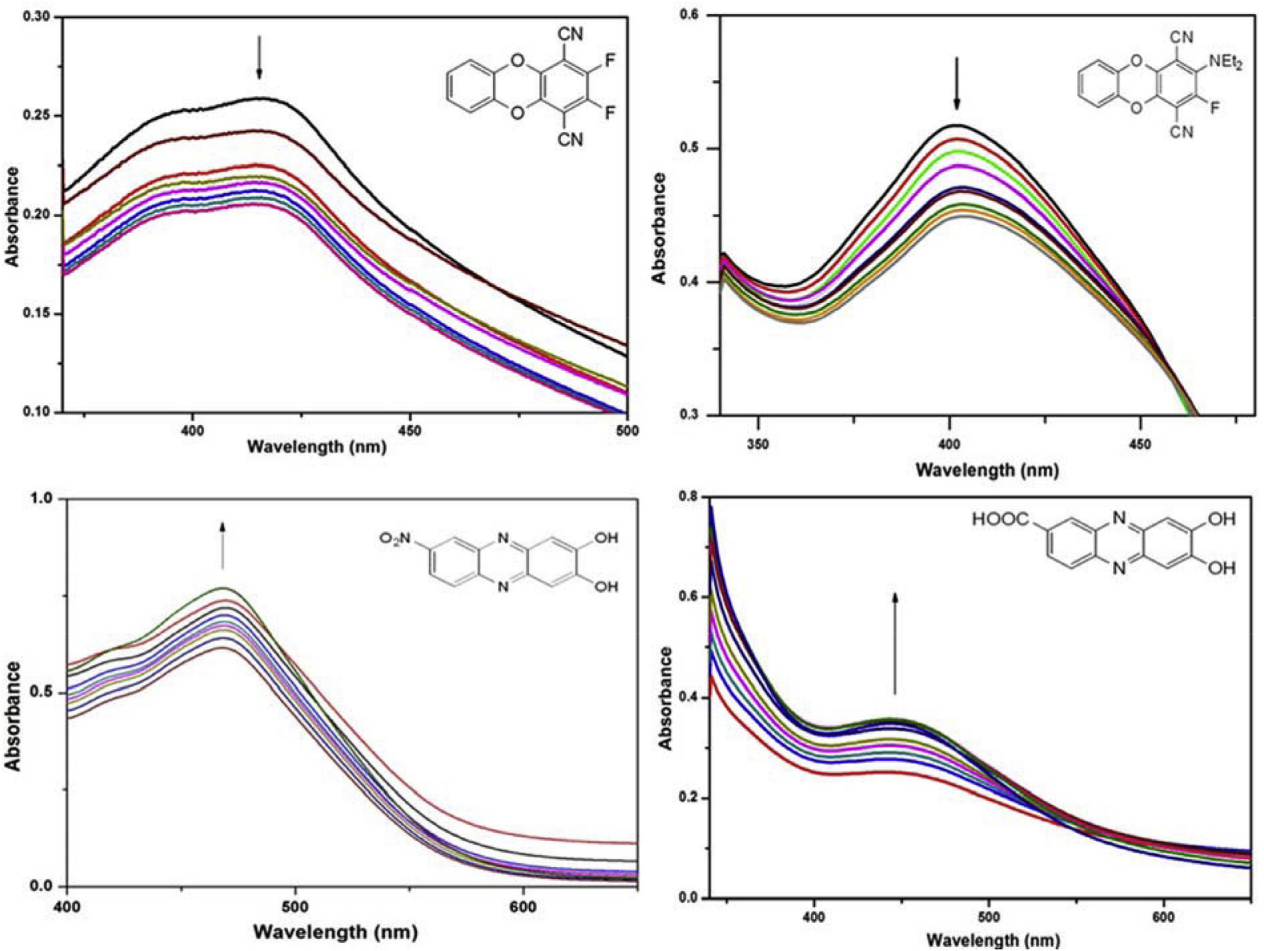
Absorption spectra of all compounds **1**–**4** in 1%DMSO in water in presence of ct-DNA. [Compound] = 5 × 10^−3^ M, stock [DNA]) = 3.5 × 10^−3^ M. Arrow indicates direction of absorbance changes upon increasing amounts of DNA.

UV–visible spectroscopy serves is one of the basic techniques to study small molecule-DNA interactions. The absorbance spectra of DNA could show hypochromism (deceased absorbance intensity) and hyperchromism (increased absorbance intensity) upon titration with varying amounts of different molecules, suggestive of molecule-DNA interactions. Hypochromic effect is found when a molecule binds to DNA promoting helix stabilization by insertion of flat aromatic species between base pairs. Hyperchromism arises from the breaking down of DNA secondary structure [19]. The intrinsic DNA-binding constants for our dibenzodioxins and phenazines were estimated by UV spectroscopy against calf thymus DNA (ct-DNA) (Fig. 4). Compound-DNA titrations were performed and spectra of compounds were recorded under variable concentrations of calf thymus DNA (Fig. 4). The result displays hypochromic effect for molecule **1** & **2** whereas for molecule **3** & **4** hyperchromic effect was observed. The intrinsic binding constants (K_b_) for the molecule–DNA adduct formation were found to be: [log_10_(K_b_)] = 5.38 for **1**, 4.97 for **2**, 5.53 for **3** and 4.92 for **4**. These values indicate moderate to strong binding interactions of compound **1**–**4** with DNA.

### 3.4 Fluorescence quenching assays

**Fig. 5:**
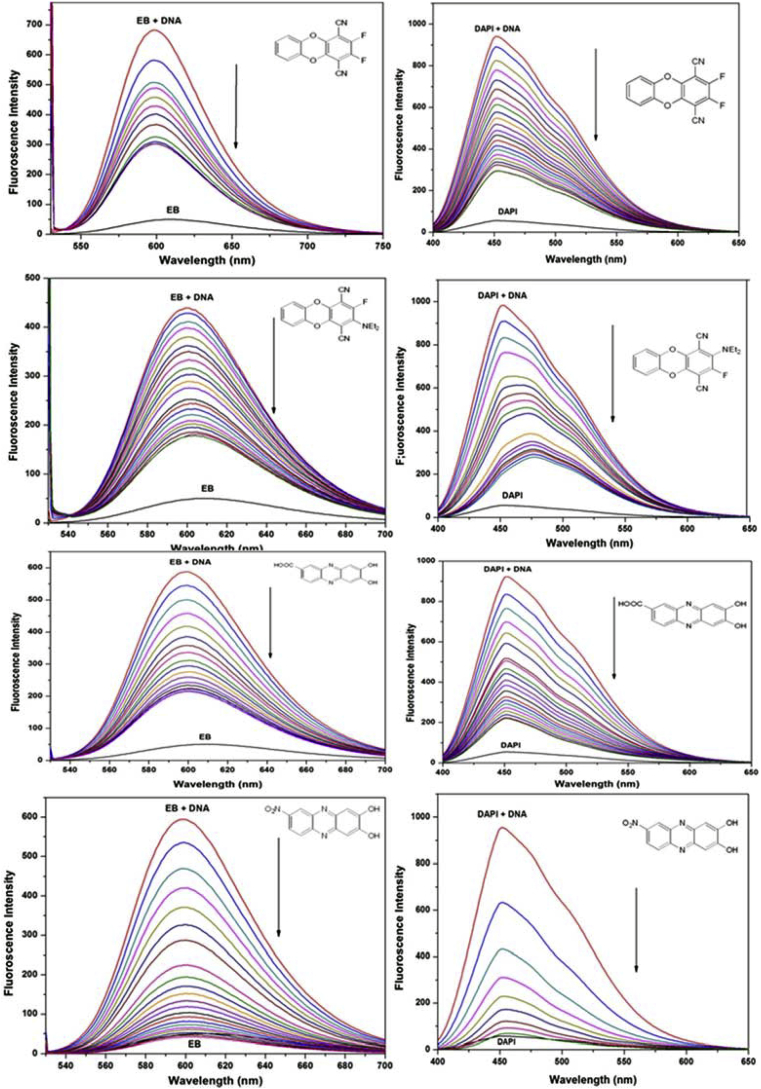
Fluorescence quenching of ct-DNA-bound EB and DAPI by **1**–**4** in tris-buffer. Dye (black line at bottom), dye–ct-DNA complex (red line at top) and dye–ct-DNA adduct titrated with various concentrations of molecules in DMSO (0–200 μM). Arrows show the direction of intensity change upon increasing compound concentrations. The concentrations of EB, DAPI, and calf thymus DNA were 2 μM, 2 μM, and 20 μM, respectively.

**Fig. 6:**
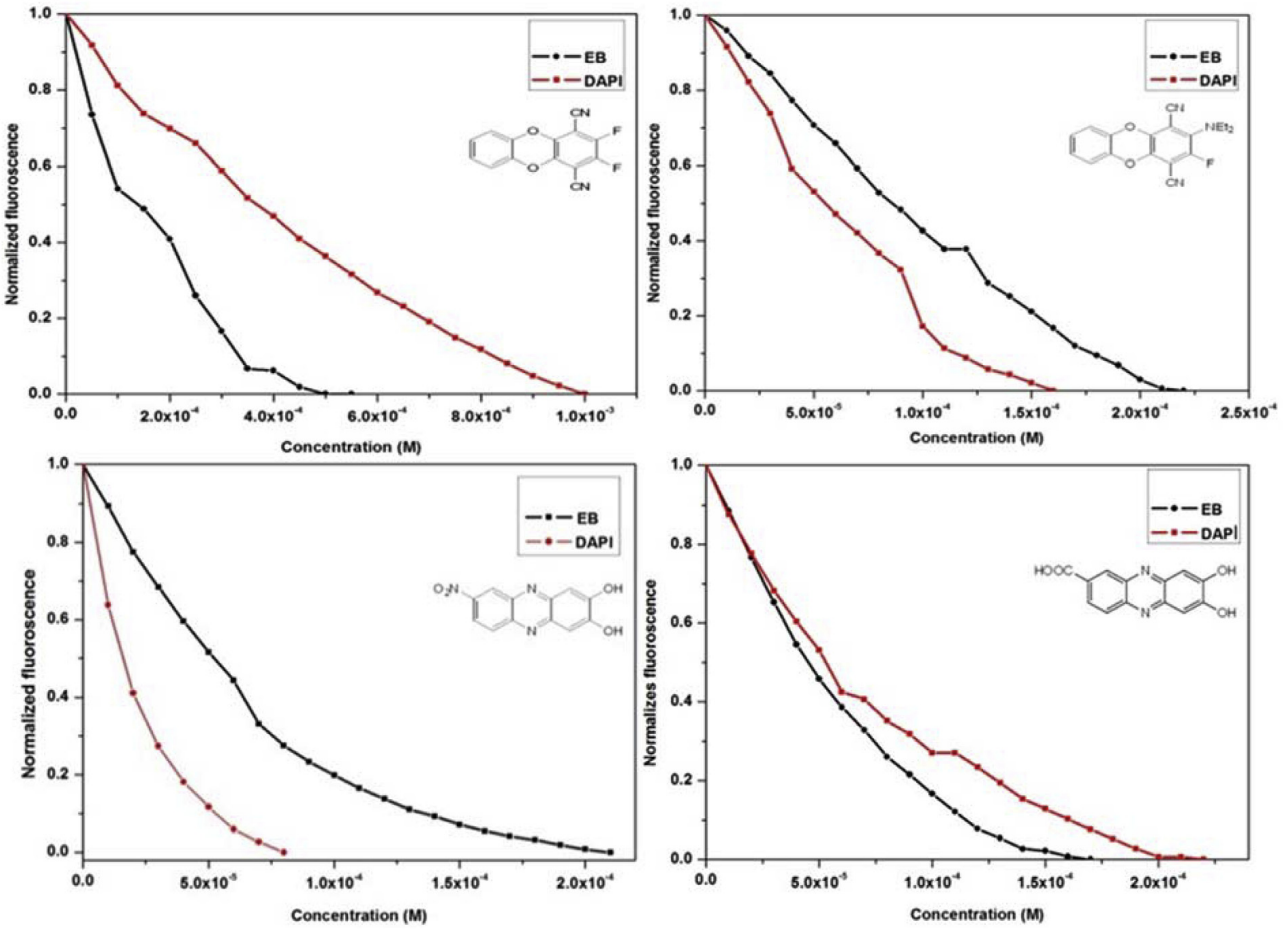
Plot of normalised fluorescence versus increasing concentration of molecules **1**–**4**.

The actual mode of binding of a molecule with DNA could be determined by competitive fluorimetric dye displacement assays. Two dyes, Ethidium bromide (EB) and 4′,6-diamidino-2-phenylindole (DAPI) with distinctly differing properties could be employed. EB is well known DNA intercalating dye which displays maximum emission at 661 nm upon binding to double stranded DNA [20]. Free molecule exhibits very low fluorescence; however, fluorescence signal magnifies when bound to DNA. DAPI is a minor groove binding molecule [21]. DAPI emission also increases approximately 30 times when excess of DNA is added. Dye–DNA binding constants were determined using dye-DNA titrations and Scatchard plots, [15] and fluorescence quenching of DNA-bound dyes were performed with our molecules **1**–**4**. Literature studies [15] revealed that displacement of a dye from its DNA-adduct is more likely by a molecule that has a similar DNA binding mode. The fluorescence spectra were recorded with incremental amounts of **1**–**4** added to the solution of DNA and EB or DAPI. Fig. 5 shows fluorescence quenching data with both the dyes. A single molecule could exhibit both minor groove binding as well as intercalative mode of binding to varying extents. Korobkova et al. [15] developed a fluorescence based method that could estimate the percentages of the two different DNA binding modes. From this method, we calculated coefficient of relative affinity to DNA (R), **1**–**4** that depends upon the efficiency of the dye (EB or DAPI) to bind to DNA and the competence of the test molecule to displace the dye from DNA. The relative affinity, R, was be presented as log[K_b(dye)_]/C_50_ where C_50_ is the concentration of **1**–**4** at 50% fluorescence quenching of a bound dye and K_b(dye)_ is DNA binding constant of bound dyes [15]. Fig. 6 represents a plot of normalised fluorescence versus concentration of our molecules (**1**–**4**). With increase in concentration, fluorescence intensity decreases. Data from Fig. 6 was used to calculate the C_50_ for our molecules (**1**–**4**). A lower value of C_50_ for a particular dye displacement (EB or DAPI) would indicate similar binding behaviour to that very dye for the test molecules **1**–**4**. The ratio of the R coefficients (RI_50_/RG_50_) where RI_50_ and RG_50_ is the affinity for intercalation (I) v/s groove binding (G) respectively is thus the ratio of two binding modes to the whole molecule–DNA adduct formation. The ratio of these two affinities gives measure of I v/s G binding modes. The values of RI_50_/RG_50_ along with I% are given in Table 1.

**Table 1:** Intercalative DNA binding mode percentages of **1**–**4**.

Compound	C_50_ (I)	C_50_ (G)	RI_50_	RG_50_	RI_50_/RG_50_	I%
**1**	1.4 × 10^−4^	3.6 × 10^−4^	4.914 × 10^4^	1.658 × 10^4^	2.97	75%
**2**	8.25 × 10^−5^	5.5 × 10^−5^	0.833 × 10^5^	1.085 × 10^5^	0.76	43%
**3**	4.50 × 10^−5^	5.5 × 10^−5^	1.528 × 10^5^	1.085 × 10^5^	1.41	58%
**4**	7.50 × 10^−5^	2.9 × 10^−5^	0.917 × 10^5^	2.058 × 10^5^	0.44	30%

Note: RI_50_/RG_50_ = R_EB_/R_DAPI_, where R_dye_ = log[K_b(dye)_]/C_50_. C_50_ is the concentration of our molecules (**1**–**4**) at 50% fluorescence quenching of EB or DAPI in molL^−1^. The I% was determined as I% = [1 + (RI_*50*_*/*RG_50_)^−1^]^−1^ x 100% [15]. K_b(DAPI)_ = 0.93 × 10^6^; K_b(EB)_ = 7.75 × 10^6^.

### 3.5 Evaluating a relative affinity and preferred binding mode

In the case of molecule **1** and **3**, the ratio RI_50_/RG_50_, were 2.97 and 1.41, respectively indicating a preference for intercalative binding for **1** and nearly equal preference for interactive and groove binding for **3** (Table 1). The ratio determined for molecule **2** and **4** were 0.76 and 0.44 respectively (Table 1). Our result suggests that intercalative binding mode (I%) of molecule **1** & **3** constitute 75% & 58% respectively to the overall binding mechanism whereas molecules **2** & **4** exhibits 43% & 30% intercalation respectively (Table 1). Hence molecules **2** and **4** are predominately groove binders.

### 3.6 Viscosity measurement studies

**Fig. 7:**
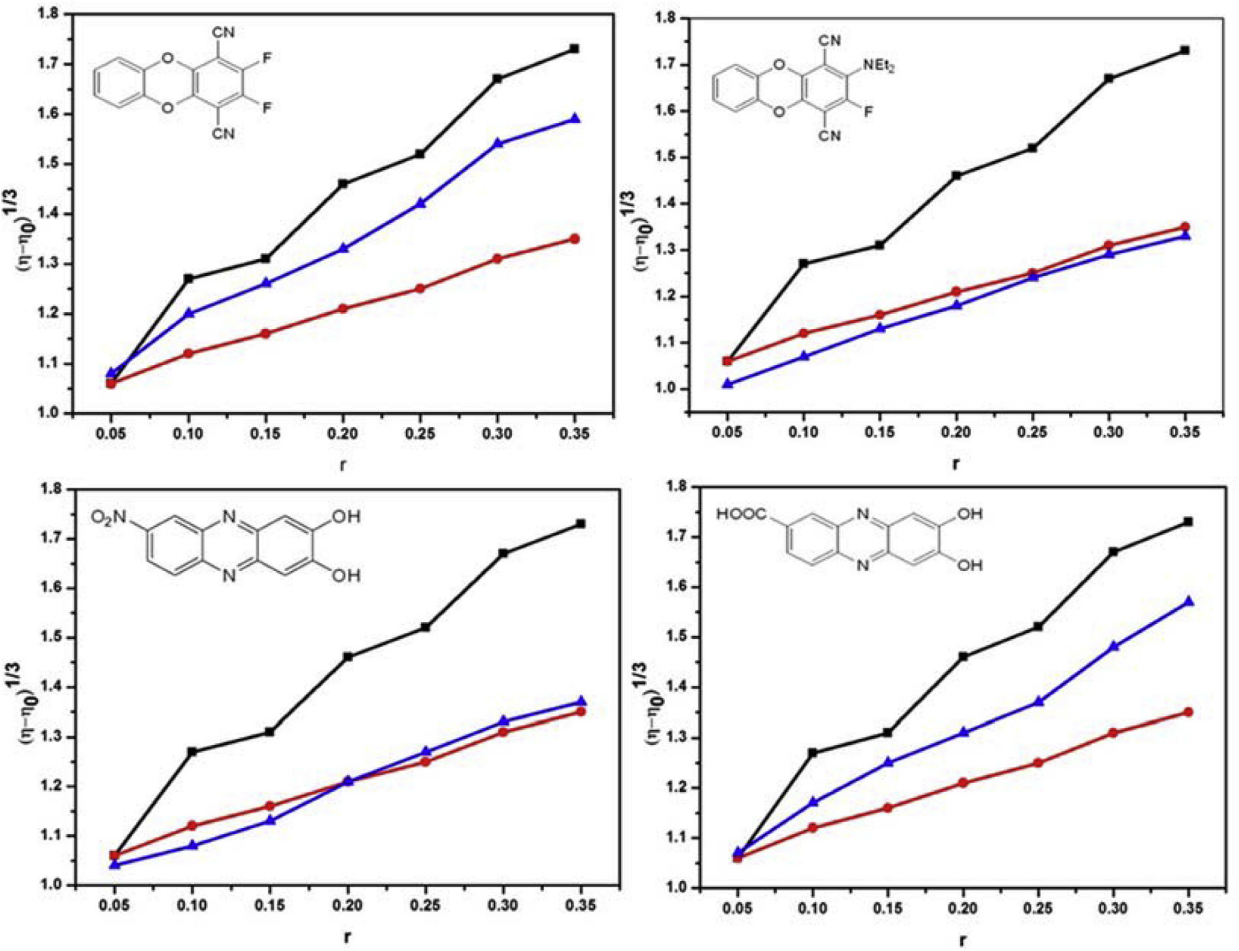
Effect of increasing amounts of EB (black line), DAPI (red line) & compounds **1**–**4** (blue line) on the relative viscosity of ct-DNA at 25 ± 0.1 °C. [DNA] = 0.5 mM, r = [Compound]/[DNA] = 0, 0.05, 0.1, 0.15, 0.2, 0.25, 0.3, 0.35 respectively.

Viscosity measurements of ct-DNA solution in absence and presence of compounds **1**–**4** were performed to further confirm the validity of results obtained from the fluorimetric method. Addition of an intercalator molecule like EB to DNA solution is expected to increase the DNA viscosity since upon molecule insertion, the base pairs separate and the DNA lengthens to accommodate the bound ligand [22]. On the other hand; a groove binder only slightly alters the DNA viscosity. As illustrated in Fig. 7, the results indicate that, the relative viscosities of the DNA solutions increases with increase in the concentrations of molecule. The increase in relative viscosity of DNA solution upon addition of EB, molecule **1** and molecule **3** was more pronounced than for DAPI, molecule **2** and molecule **4**. Upon increasing the amounts of molecules **1** & **3**, the relative viscosity of DNA increases steadily similar to the behavior of EB. In contrast, DNA viscosity increases only slightly similar to the behavior of DAPI. Based upon net change in viscosity (Δη) (Table 2) the molecules may arranged in the following decreasing order of viscosity change: EB > **1** > **3** > **4** ∼ **2** > DAPI. These results suggest that the preferred mode of DNA binding by **1** is intercalation, molecule **3** has nearly equal preference for both intercalative and groove binding, whereas molecules **2** & **4** exhibits preference for minor grove binding. Thus, the viscosity results are in agreement with the finding of the fluorescence studies.

**Table 2:** Net change in viscosity (η) of ct-DNA solution induced by addition of EB, DAPI and compounds **1**–**4**.

Compounds	Δ η^1/3^
EB	0.67
DAPI	0.29
**1**	0.51
**2**	0.32
**3**	0.50
**4**	0.33

## 4 Conclusion

Design and synthesis of small planar aromatic molecules that can recognize specific sites of DNA for binding through adduct formation is an important area of current interest. In this direction, we have previously designed and synthesised planar heteroaromatic molecules like dibenzodioxins and phenazines and here studied their interaction with ct-DNA using various biophysical techniques. The binding ability of our synthesised molecules with ct-DNA at various concentrations is investigated by UV-absorption and fluorescence spectroscopy. Effects of such binding on the viscosity of DNA are also presented in this paper. The viscosity experiments and competitive fluorescence based dye displacement studies confirmed the DNA binding preference for all experimental molecules (**1**–**4**). The results suggested that molecule **1** has preference for intercalative binding, **2** and **3** have nearly equal and opposite preferences for both intercalation as well as groove binding, whereas molecule **4** prefers minor grove binding mode. The observed binding preferences for molecules **1**–**4** could be related to their subtle structural differences. For a molecule to bind with DNA via intercalative mode it should ideally have ‘anthracene-like’ flat structure with no functional groups capable of disrupting its overall planarity. For groove binding, planar molecules should also possess hydrogen bond donating and accepting functional groups, which could establish non-covalent interactions with the A-T rich regions lying along the minor grooves of DNA. In this regard our molecule **1** is very much ‘anthracene-like’ with no disruption of planarity by other groups, whereas as molecule **2** contains the diethylamino moiety which could interfere with its overall planarity from a base-pair stacking point of view and reduce its intercalative binding percentage. For molecules **3** and **4** despite their good planar structure they also have hydrogen bond donating and accepting groups at their peripheral ends. In addition, the central nitrogen atoms of the pyrazine ring in both **3** and **4** could act as a mimic of the central nitrogen atom in the DAPI structure, where it acts as an H-bond acceptor, when bound to the minor groove of DNA [23]. Therefore based on the diverse structural features of **1**–**4** a possible explanation for their binding preferences could be found. The results of these binding studies will serve as a convenient guide in designing new promising anticancer agents.
